# Brazilian version of the Brief Male Sexual Function Inventory (BSFI) in adult men: cultural adaptation and measurement properties

**DOI:** 10.1093/sexmed/qfaf029

**Published:** 2025-05-12

**Authors:** Erisvan Vieira da Silva, Guilherme Tavares de Arruda, Eduarda Brollo Berni, Amanda dos Santos Candido, Hedioneia Maria Foletto Pivetta, Melissa Medeiros Braz

**Affiliations:** Federal University of Santa Maria, Postgraduate Program in Movement Sciences and Rehabilitation, Santa Maria, Brazil; Federal University of São Carlos, Postgraduate Program in Physiotherapy, São Carlos, Brazil; Federal University of Santa Maria, Department of Physiotherapy and Rehabilitation, Santa Maria, Brazil; Federal University of Santa Maria, Department of Physiotherapy and Rehabilitation, Santa Maria, Brazil; Federal University of Santa Maria, Postgraduate Program in Movement Sciences and Rehabilitation, Santa Maria, Brazil; Federal University of Santa Maria, Postgraduate Program in Movement Sciences and Rehabilitation, Santa Maria, Brazil

**Keywords:** sexual behavior, psychometrics, patient outcome assessment, sexual health

## Abstract

**Background:**

There is a need for a more comprehensive tool for assessing male sexual function that is inclusive of diverse gender identities and sexual orientations.

**Aim:**

Culturally adapt the Brazilian version of the Brief male Sexual Function Inventory (BSFI) and evaluate its measurement properties in adult men.

**Methods:**

We conducted an online survey with 727 Brazilian men over 18 years of age (31.73 ± 10.88 years). According to COnsensus-based Standards for the selection of health Measurement INstruments (COSMIN), we translated and evaluated the content validity, structural validity, internal consistency, construct validity, test–retest reliability, measurement error, and minimum and maximum effects of the BSFI.

**Outcomes:**

The final Brazilian version of the BSFI consisted of 10 items and was found to be relevant, comprehensive, and comprehensible.

**Results:**

Although it showed good content validity and reliability, the BSFI showed inadequate structural validity and insufficient internal consistency for at least one factor.

**Clinical Implications:**

The Brazilian version of the BSFI can be used in adult males in community samples, pending evaluation in clinical samples.

**Strengths and Limitations:**

The sampling bias associated with online recruitment may have excluded individuals without access to the Internet, thereby compromising the representativeness of the sample. This may limit generalizability. However, COSMIN guidelines were followed to ensure methodological rigor. The BSFI adaptation provides a Patient-Reported Outcome Measure for assessing male sexual function, but has limitations in structural validity and internal consistency.

**Conclusion:**

The Brazilian version of the BSFI showed sufficient validity (content validity and hypothesis testing for construct validity) in adult men and sufficient test–retest reliability.

## Introduction

Sexual dysfunctions emerges when compromised sexual function causes discomfort or distress, traditionally categorized into four distinct groups: lack of desire or interest, arousal dysfunction, orgasm and/or ejaculation dysfunction, and pain during sexual activities.^1^^,^^2^ Impairment of sexual function leads to mood disorders, decreased sexual satisfaction, and a reduction in the quality of marital relationship.^3^ The global prevalence of sexual dysfunctions in men has not been reported. However, some studies and reviews have reported a prevalence of sexual dysfunctions, particularly erectile dysfunction (ED), ranging from 31% to 95%.^4-6^ Therefore, the assessment of male sexual function is essential for the appropriate treatment of patients. In addition, with the progressive increase of sexual dysfunctions in men, appropriate assessment approaches are essential.

While ED can be measured through various methods, such as nocturnal penile monitoring, imaging techniques, neurological studies, and psychological assessment,^7^ the sexual function is more reported by the patients themselves.^8^ In this context, Patient-Reported Outcome Measure (PROM) has been suggested as a valuable approach to assess sexual function and measure ED.^9^ Among the PROMs currently available, the International Index of Erectile Function (IIEF) is the most widely used PROM for assessing erectile function due to its comprehensiveness and widespread acceptance in both research and clinical practice.^10^ However, the IIEF has some limitations. In its development study, the authors excluded other domains of the sexual response cycle (eg orgasm/ejaculation) in which the patient’s sexual function is not fully assessed. In addition, the IIEF was developed specifically as a measure to assess response to ED treatment and was designed exclusively for cisgender and heterosexual men and presupposes vaginal intercourse.^11^ Given this, there is a need for the use of a PROM that encompasses issues related to other sexual dysfunctions and includes individuals of diverse gender identities and sexual orientations. In this regard, O’leary et al.^8^ developed the Brief Male Sexual Function Inventory (BSFI).

The BSFI is a reflective model (ie the construct is manifested in the items) PROM that can be quickly filled out and scored to assess male sexual function.^3^ This PROM assesses five domains of male sexual function, including sexual drive, erection, ejaculation, perceptions of problems in each area, and overall satisfaction. Items are scored on a 5-point Likert scale, ranging from 0 to 4, and the total score is calculated as the average score relative to each domain.^8^ In the BSFI development study, its measurement properties (internal consistency and test–retest reliability) were adequate.^8^ Adequate internal consistency values for the BSFI were also reported in Norway with a single factor^12^ and in the Persian version with five factors structure.^3^

The BSFI offers significant advantages in clinical practice by providing a comprehensive and easy-to-use instrument for assessing male sexual function. Although the IIEF is widely used and validated for assessing erectile function, the BSFI assesses multiple domains, including sexual drive, erection, ejaculation, perception of problems in each domain, and overall satisfaction. While the IIEF is primarily designed to measure treatment response in ED, it does not include other critical aspects of male sexual function such as ejaculation and overall sexual satisfaction, limiting its ability to provide a complete assessment in clinical practice. In addition, the IIEF was developed exclusively for cisgender and heterosexual men and assumes vaginal intercourse as the primary sexual activity,^10^ limiting its applicability to individuals with diverse gender identities and sexual orientations. In contrast, the BSFI addresses these limitations by providing a broader assessment of male sexual function beyond erectile function, taking into account different domains of the sexual response cycle. In addition, the BSFI makes no assumptions about gender identity or sexual orientation, making it a more inclusive instrument for clinical and research settings. Its ease of administration and scoring further enhances its utility, facilitating its use in both clinical practice and research. Thus, the BSFI provides a valuable alternative PROM to existing measures, allowing for a more comprehensive and inclusive assessment of male sexual function in diverse populations. In addition, the BSFI has not been culturally adapted for the Brazilian population. Its cultural adaptation and evaluation in Brazil will aid healthcare professionals in appraising male sexual function in both clinical settings and scientific research. We hypothesize that the Brazilian version of the BSFI will have sufficient measurement properties in adult men. Therefore, the aims of this study were to culturally adapt the Brazilian version of the BSFI and evaluate its measurement properties in adult men. Specifically, does the Brazilian version of the BSFI demonstrate sufficient measurement properties when applied to adult men?

## Methods

### Design

This is a cross-sectional, repeated-measures study reported according to the COnsensus-based Standards for the selection of health Measurement INstruments (COSMIN)^13^^,^^14^ to assess the measurement properties of the BSFI online version in adults. This study was approved by the Institutional Ethics Committee (n. 5.489.920) and was conducted online from June 2022 to November 2023. The follow measurement properties were evaluated: content validity (degree to which a measuring instrument really seems to be an adequate reflection of the construct to be measured), structural validity (degree to which the scores of an instrument are an adequate reflection of the dimensionality of the construct to be measured), internal consistency (degree of interrelation between items), hypothesis testing for construct validity (degree to which an instrument’s scores are consistent with hypotheses based on the assumption that the instrument validly measures the construct to be measured), test–retest reliability (degree to which a measurement is free of measurement error using different sets of items over time) and measurement error (systematic and random error of a patient’s score that is not attributed to real changes in the construct to be measured).^15^ The floor and ceiling effects were also assessed.

### Procedures and population

Participants were invited to participate in the study through Facebook, Instagram, WhatsApp, and emails from universities through a Google Forms link. We used convenience sampling to include all Brazilians over 18 years old and able to speak, read and write in Brazilian Portuguese. We included cisgender men, from different geographic regions of Brazil, age, skin color, and education to increase socio-cultural variability. Cisgender men with a medical diagnosis or self-report of mental illness and schizophrenia were excluded. The sample size for structural validity was calculated according to the COSMIN recommendations^16^: 7 to 10 men per item of the PROM, but >100 men is adequate.

### Cultural adaptation and content validity assessment

We culturally adapted and assessed the content validity of the BSFI in five steps: direct translation, synthesis of the translation, back-translation, expert committee review, and final version testing with the Brazilian adult men (target population). Firstly, two independent translators translated the original version of the BSFI into Brazilian Portuguese. Both translators had Brazilian Portuguese as their mother tongue and had no knowledge about the BSFI. The two translated versions of the BSFI were synthesized into a single version and independently back-translated into English by two other Native American translators. The two translators did not know the original instrument. Discrepancies between back-translations were resolved by consensus. Following these steps, the content validity of the BSFI was assessed by an expert committee of eight professionals with expertise in human sexuality and/or experience in studies of the measurement properties of PROMs. Finally, the content validity of the BSFI was assessed by the target population and culturally adapted into Brazilian Portuguese.

The content validity of the BSFI was assessed by the expert committee and the target population through semi-structured interviews via Google Meet. The interviews were conducted by a trained interviewer using an interview guide that included topics related to concepts and assessment objectives, missing concepts, and suggestions for modifying the PROM. The interviews were recorded, literally transcribed, and content analyzed by two other researchers. We asked the expert committee and the target population to rate the relevance and comprehensibility of the instructions, recall time, response options, and items of the BSFI to assess sexual function in a general population of Brazilian men, as well as the comprehensiveness of the BSFI items. A spreadsheet was used to control for response saturation after transcribing the interviews. The content of the interview transcripts was analyzed by content analysis and the main categories of concepts and words were described. Data from the interviews were analyzed independently by two researchers.^17^

### Test–retest reliability and measurement error

Following the COSMIN recommendations,^16^ we used 14 days as the time interval to assess the test–retest reliability and measurement error. To check that participants did not undergo sex therapy or any intervention in the retest, they should answer “yes” or “no” to the question “Since the first time you participated in this research until today, have you had any sexual or sexuality therapy?”. Participants who answered “no” to this question on the retest were included in the analysis of test–retest reliability and measurement error.

### Measures

#### Sample characterization

Participants were characterized by a questionnaire consisting of the following items: age, region of residence in Brazil (Southeast, Northeast, South, North, or Midwest), education (primary or secondary, and higher education), sexual orientation (heterosexual, gay, bisexual, and other), anxiety and/or depression diagnosed by clinical exams, and relationship (no sexual partner, or with sexual partner). For the sexual orientation, we included pansexual, asexual, and demisexual in the “other” category.

#### Brief male Sexual Function Inventory

The BSFI is a reflective PROM consisting of 11 items divided into five domains (sexual drive, erection, ejaculation, sexual problems, and overall sexual satisfaction) that assess male sexual function. BSFI items are answered on Likert scales ranging from 0 (or 1 point) to 4 (or 5 points). Scores are calculated for each of the five domains, along with a total BSFI score. The lower the total score, the greater the impairment of sexual function, and higher scores (closer to 45) are considered to indicate little or no sexual dysfunctions.^8^

#### International Index of Erectile Function

The IIEF consists of 15 items covering five domains of sexual function: erectile function, orgasm, sexual drive, sexual satisfaction, and overall satisfaction. Each item is scored from 1 (very low/almost never or never/extremely difficult) to 5 (very high/almost always or always/not difficult), and the final score for each domain is calculated by summing the responses, with higher scores indicating better sexual function.^9^ Gonzáles et al.^18^ validated this instrument for Brazilian Portuguese with adequate internal consistency (α = 0.89) and excellent test–retest reliability (*r* > 0.75).

### Data analysis of other measurement properties

A qualitative approach was used to analyze the data for content validity to capture the nuanced perspectives of the expert committee and the target population, as it allowed for in-depth exploration and thematic categorization of their responses. Content validity was assessed by the responses of the expert committee and the target population through content analysis with selective coding of transcripts and grouping of codes into thematic categories. In the content analysis, we noted the frequency of concepts and words contained in the data. In the end, the categories of concepts and words were described.^17^ Consensus was reached between the experts and the target population when no new suggestions for modification of the instrument were received from them.

Structural validity was assessed by exploratory factor analysis (EFA) and confirmatory factor analysis (CFA). In EFA, Kaiser-Meyer-Olkin (KMO) test and the Bartlett sphericity test were used to assess the factorability of the data. KMO ≥ 0.70 and *P* ≤ 0.05 in the Bartlett sphericity test indicated criterion to perform exploratory factor analysis. We performed EFA on the total sample using Weighted Least Squares with Mean and Variance adjusted (WLSMV) as the estimator and multiple extraction methods, including Parallel Analysis, to retain the number of factors due to the ordinal response options of the BSFI items. If necessary, items with factor loading <0.40 were excluded.^19^ For the CFA, the total sample was split equally and randomly in R version 4.2.1 (R Core Team) and RStudio version 2024.04 (Posit Software, PBC) to check whether the factor structure is similar in a subsample. We used the WLSMV as the estimator, Root Mean Square Error of Approximation (RMSEA), Comparative Fit Index (CFI), and Tucker-Lewis Index (TLI). RMSEA <0.08, CFI and TLI > 0.90 were considered the appropriate model. RMSEA values between 0.08 and 0.10 indicated a mediocre fit.^20^ When possible, we used the Bayesian Information Criterion (BIC) to compare the models in this study. The model with the lowest BIC was considered the most appropriate. Items with modification indices (MI) > 40 000 had residual covariances. Based on the developmental study of the BSFI and changes during the content validity assessment, we compared the model suggested by the EFA with a one-factor model and a second-order model. Each factor included items representing the five domains of the BFSI (ie sexual drive, erection, ejaculation, sexual problems, and overall sexual satisfaction). For the second order model, we assume that there is a higher factor that contains the five factors and that can explain sexual function. Internal consistency was assessed using McDonald’s Omega (Ω). Values of Ω ≥ 0.70^21^ were considered adequate.

For the BSFI total score, all items were summed (item 1 + item 2 + … + item 10). The distribution of the BSFI total score was assessed by floor and ceiling effects. Floor or ceiling effects less than 15% were considered appropriate.^16^

The hypothesis testing for construct validity was assessed using Pearson’s correlation between BSFI total score and IIEF total score. The correlation magnitude followed Cohen’s criteria^22^: weak (*r* < 0.30), moderate (*r* = 0.30 to 0.50) and strong (*r* > 0.50). We hypothesized a positive and strong correlation between the BSFI total score and the IIEF total score, as both instruments measure the same construct.

We assessed test–retest reliability using the intraclass correlation coefficient (ICC_agreement_) with a two-way mixed-effects model with interaction for absolute agreement between mean measurements. An ICC_agreement_ ≥ 0.7 was considered adequate.^23^ Measurement error was calculated with the Standard Error of Measurement (SEM_agreement_) using the formula [SD_difference_/√2], the Smallest Detectable Change (SDC_agreement_) at the individual level using [SEM*1.96*√2],^16^ and the Limits of Agreement (LoA) using the formula [d- ± (1.96 × SD_difference_)]. The d- and SD_difference_ are the mean and standard deviation (SD) of the differences between the test and retest scores, respectively.^24^

Missing values were excluded from analyses (sample characterization variables). Responses to the BSFI were mandatory and had no missing values. All analyses were conducted in lavaan package in R version 4.2.1 (R Core Team) and RStudio version 2024.04 (Posit Software, PBC).

## Results

### Content validity

The content validity of the BSFI was assessed by a committee of seven experts through cognitive interviews with nine cisgender men after the translation process. The content validity of the BSFI, including face validity, was considered comprehensible, comprehensive, and relevant by the expert committee. However, some changes were suggested. The expert committee suggested changing the response options for item 2 from “médio-alto/medium-high” and “alto/high” to “alto/high” and “máximo/maximum”, and for item 11 from “neutro ou misto/neutral or mixed” to “neutro/neutral”. Item 7 was excluded because the amount of semen does not characterize sexual dysfunction. The order of the items was reorganized so that the items related to each domain of sexual function were closer together. After the modifications, a new phase was carried out with the expert committee, which considered the modified version of the instrument to be appropriate.

In the cognitive interview phase with cisgender men (*n* = 9), participants had a mean age of 28.78 ± 9.05 years and the majority were from the southern region of Brazil (*n* = 5; 55.55%), had sexual activity in the last 4 weeks (*n* = 8; 88.89%), and were heterosexual (*n* = 5; 55.55%) and homosexual/gay (*n* = 4; 44.45%). Participants found the BSFI instructions, recall time, items, and response options comprehensible, comprehensive, and relevant. The final translated version of the BSFI into Brazilian Portuguese is presented in Appendix A.

### Sample characteristics

In the total sample, 727 men (31.73 ± 10.88 years) participated in the study. Table 1 shows the socio-demographic characteristics of the participants. The majority of participants lived in the South of Brazil (*n* = 253; 34.80%), had higher education (*n* = 715; 98.35%), were heterosexual (*n* = 429; 59.09%), and had a sexual partner (*n* = 449; 61.76%).

**Table 1:** Characteristics of the study participants.

**Characteristics**	**Total sample (*n* = 727)**	**Split sample (*n* = 366)**
Age (years), mean ± SD	31.73 ± 10.88	31.33 ± 10.82
Geographic region^a^, *n* (%) South Southeast Midwest North Northeast	253 (34.80) 187 (25.72) 42 (5.79) 39 (5.36) 206 (28.33)	132 (36.06) 86 (23.50) 19 (5.20) 22 (6.01) 107 (29.23)
Education, *n* (%) Primary or secondary Higher education	12 (1.65) 715 (98.35)	09 (2.46) 357 (97.54)
Sexual orientation^a^, *n* (%) Heterosexual Gay Bisexual Other	429 (59.09) 161 (22.18) 97 (13.36) 39 (5.37)	222 (60.66) 125 (34.15) 10 (2.73) 09 (2.46)
Relationship, *n* (%) No sexual partner With sexual partner	278 (38.24) 449 (61.76)	136 (37.16) 230 (62.84)
Anxiety^a^, *n* (%)	165 (22.70)	80 (21.86)
Depression^a^, *n* (%)	75 (10.32)	38 (10.38)

SD: Standard deviation. ^a^: Missing value.

### Structural validity and internal consistency

For the total sample (*n* = 727), KMO (0.738) and Bartlett’s sphericity test (ꭓ^2^ = 2968.62; df = 45; *P* < 0.001) were adequate for factorization. Factor analysis suggested a one- and 5-factor structure, respectively, with 59% and 35.2% of the variance explained for the first factor and 15.4%, 8.4%, 4.8%, and 3.3% for the subsequent factors. Items in the 5-factor were divided as follows: factor 1 (Sexual drive: items 1 and 2), factor 2 (Sexual drive problem and Erection: items 3, 4, and 7), factor 3 (Erection function: items 5 and 6), factor 4 (Ejaculation: items 8 and 9), and factor 5 (Sexual satisfaction: item 10). Since factor 5 contains only one item and has the highest correlation with the factor compared to the other factors (*r* = 0.213), item 10 from factor 5 was added to factor 2 for CFA. The new 4-factor model contains: factor 1 (Sexual drive: items 1 and 2), factor 2 (Sexual drive problem, erection, and sexual satisfaction: items 3, 4, 7, and 10), factor 3 (Erection function: items 5 and 6), and factor 4 (Ejaculation: items 8 and 9). Table 2 compares the factor loadings, fit measures, and internal consistency of all models (one-factor, 4-factor, and second-order with 4 factors) for the split sample (*n* = 366). All models had some limitations. The one-factor model had low factor loadings (<0.400) for items 10, and inadequate RMSEA (0.119). Residual covariances were added between items 1 and 2 (0.73), 1 and 4 (−0.41), 1 and 7 (−0.42), and 2 and 7 (−0.35). Item 10 in the 4-factor and second-order models also had low factor loadings (<0.400). In addition, both models had inadequate RMSEA (4-factor: 0.096, second-order: 0.116) and not sufficient McDonald’s Omega for factor 4 (0.585). Therefore, no BSFI model was adequate.

**Table 2:** Factor loadings, fit measures, and internal consistency for items in the Brazilian version of the BSFI.

Items	One-factor	4-factor	Second-order (4-factor), F: 0.423, F2: 0.815, F3: 0.958, F4: 0.774
1. Days of sexual drive	0.415	F1: 0.834	F1: 0.809
2. Level of sexual drive	0.419	F1: 0.928	F1: 0.956
3. Sexual drive as a problem	0.488	F2: 0.539	F2: 0.537
4. Difficulty getting an erection	0.776	F2: 0.820	F2: 0.822
5. Frequency of a partial or full erections	0.685	F3: 0.716	F3: 0.711
6. Frequency of hard erections for intercourse	0.815	F3: 0.909	F3: 0.916
7. Problems maintaining an erection	0.784	F2: 0.810	F2: 0.808
8. Difficulty ejaculating	0.549	F3: 0.695	F3: 0.715
9. Problems with ejaculation	0.468	F4: 0.595	F4: 0.579
10. Satisfaction with sex life	0.312	F2: 0.336	F2: 0.340
ꭓ^2^(df)	191.545(31)	125.924(29)	184.204(31)
CFI	0.917	0.950	0.921
TLI	0.879	0.922	0.885
RMSEA (90%CI)	0.119 (0.103– 0.136)	0.096 (0.079– 0.113)	0.116 (0.100– 0.133)
BIC	10228.087	-	-
McDonald’s Omega (Ω)	0.823	F1: 0.872, F2: 0.741, F3: 0.789, F4: 0.585	General factor: 0.823, F1: 0.872, F2: 0.741, F3: 0.789, F4: 0.585

Abbreviations: BSFI: Brazilian version of the Brief Male Sexual Function Inventory. BIC: Bayesian Information Criterion. CFI: Comparative Fit Index. RMSEA: Root Mean Square Error of Approximation. TLI: Tucker-Lewis Index.

### BSFI floor and ceiling effects

In the current study, the BSFI total score was calculated for the total sample (*n* = 727, 31.14 ± 5.86 points). There were no floor and ceiling effects in the total sample (0.14% minimum and 0.14% maximum score).

### Hypothesis testing for construct validity

As we expected, correlations between BSFI total score (31.14 ± 5.86 points) and IIEF total score (54.41 ± 19.50 points) were positive and strong (*r* = 0.477; *P* < 0.001) for the total sample.

### Test–retest reliability and measurement error

One hundred and ninety-three (26.55%) men responded to the retest. However, 159 (21.87%) from the total sample did not receive any sexual therapy or intervention between test and retest and responded to the retest between 12 and 22 days. The test–retest reliability value was considered adequate (ICC = 0.843; 95%CI 0.785–0.885), and the mean difference (d-) between the test and retest was 0.50 point. For the measurement error, SEM_agreement_, SDC_agreement_, LoA_inf_, and LoA_sup_ were, respectively, 2.93 (95%CI −2.81–8.66), 8.09 (95%CI −7.76–23.93), −2.26 and 3.26.

## Discussion

In this study, we culturally adapted and evaluated the measurement properties of the Brazilian version of the BSFI in adult men. Suggestions from the expert committee led to adaptations of the response options, the exclusion of item 7, and a reordering of the items to better reflect the domains of sexual function. After the modifications, the final version of the BSFI consisted of 10 items and was considered relevant, comprehensive and comprehensible by the expert committee and the target population. However, its structural validity and internal consistency were insufficient.

The CFA results of the BSFI models were not sufficient to explain the factor structure of the instrument. Furthermore, all models showed low factor loadings for item 10, suggesting that this item may not adequately represent the underlying constructs of male sexual function. In the studies by Myckletun et al.^12^ and Vallejo–Medina, Guillén–Riquelme and Sierra,^25^ an analysis of the unidimensionality of the scale was performed, but the results also did not allow a definitive conclusion. In addition, the internal consistency of factor 4 of the BSFI was not sufficient. However, it is important to highlight that the BSFI is a short measure and as such presents challenges when evaluated using CFA and EFA. According to the COSMIN guidelines,^26^ structural validity is an essential aspect of assessing the degree to which the scores of the instrument adequately reflect the dimensionality of the construct being measured. However, short measures are inherently unsuitable for dimensionality reduction techniques because they capture multiple facets of a construct with minimal items.

While the BSFI offers practical advantages, such as faster administration compared to the IIEF, it may not show sufficient fit measures of structural validity. A possible reason for this in the BSFI is the need for more items to assess male sexual function in general (eg inclusion of items on satisfaction, sexual drive, and orgasm) such as those present in the IIEF. Poor model fit and low item saturation are often observed in short measures when tested under one-factor solutions. Furthermore, when tested in multi-factor models, factors typically load on only one or two items, raising concerns about statistical under-identification.^27^ These expected results have also been observed in previous studies using the BSFI.^12^^,^^25^ Similarly, assessing internal consistency in brief measures presents unique challenges. Because brief instruments often contain items that assess different facets of a construct, the intercorrelations among the items may not be high, which naturally results in lower Cronbach’s alpha values. In addition, when Cronbach’s alpha is calculated separately for subscales containing only two or three items, the values tend to be moderate or low.^16^^,^^28^ This is partly due to the way in which Cronbach’s alpha is affected by the number of items included in the calculation-large numbers of items inflate the coefficient, while small numbers deflate it.^16^^,^^28^ Given these considerations, the lower than sufficient internal consistency values found in this study are not surprising and should not necessarily be interpreted as an indication that the BSFI lacks utility. Thus, while the BSFI does not show sufficient structural validity and internal consistency, these limitations should be viewed in the context of its design as a brief measure. Therefore, while the BSFI should be used with caution, particularly in clinical settings where more comprehensive assessments may be needed, it remains a potentially useful instrument for assessing male sexual function, especially in non-clinical populations where brevity is a priority.

For construct validity, our first hypothesis was to find a positive and strong association between the BSFI and the IIEF, and this hypothesis was confirmed. The IIEF and BSFI are PROMs that have been widely used for clinicians and researchers to assess male sexual function.^10^^,^^29^^,^^30^ Both instruments address relevant domains of sexual function, including erectile function, satisfaction with intercourse, orgasm, sexual drive, and overall satisfaction.^8^^,^^9^ However, the relationship between the IIEF and BSFI scores has not been examined in previous studies. Our study found that the higher a man’s BSFI score, the higher his IIEF score. This pattern may be explained by the fact that both instruments address related aspects of male sexual function. However, this result was not sufficient to recommend the use of the BSFI for the assessment of male sexual function.

The test–retest reliability results of the BSFI indicated adequate statistics over time. This suggests that the participants remained stable over time for the construct measured. However, when considering measurement error, although higher SEM and SDC scores were observed, there is still some variability in scores between tests that must be taken into account when interpreting results over time. The assessment of measurement error helps to minimize systematic and random errors from real changes in the measured construct.^16^ Perhaps this was not observed in the BSFI measurement error results due to some bias that we cannot explain.

## Limitations

Based on the characteristics of the target population that participated in the content validation and the general population that participated in the evaluation of the other measurement properties, several limitations were identified. The sampling bias associated with online recruitment may have excluded individuals without access to the Internet, thereby compromising the representativeness of the sample. In addition, there were only cisgender participants, with a prevalence of young, higher education, heterosexual, and living in the southern region of Brazil, which may limit the generalizability of the results to more diverse populations. Given that sexual dysfunction generally increases with age,^31^ the absence of older adults in the sample may have biased the assessment of the BSFI. Because the BSFI development study^8^ included two groups of men with mean ages of 41 and 60 years, the measurement properties of the BSFI should be evaluated in populations that include middle-aged and older individuals to ensure a more comprehensive assessment of its measurement properties. Thus, the underrepresentation of older individuals in this study limits the generalizability of the results. Future research should consider more age-diverse samples to better capture the full spectrum of sexual function across age groups.

Another limitation of this study is the use of only the IIEF for hypothesis testing for construct validity. Although the IIEF is a recognized instrument that assesses a similar construct (ie male sexual function),^18^ the inclusion of additional measures (eg sexual or general life satisfaction, well-being, or anxiety/depression) could have provided a broader assessment of the construct validity of the BSFI. The use of a single external measure, particularly one that assesses a closely related construct, may have limited the ability to capture different aspects of male sexual function. Future studies should include a more diverse set of external criteria to strengthen the validation process and provide a more comprehensive understanding of the measurement properties of the BSFI. However, the study has strengths in following the COSMIN guidelines.^13^^,^^14^^,^^16^ This demonstrates methodological rigor in assessing the measurement properties of the instrument and its reporting, which increases the reliability of the results.

The cultural adaptation of the BSFI provides researchers and clinicians with another PROM to assess male sexual function in adult men. However, the lack of structural validity and insufficient internal consistency may limit its applicability in research and clinical practice. Considering these aspects, it is essential to interpret the results with caution, recognizing both the limitations and the strengths of the study and contextualizing them appropriately. We recommend that future studies explore alternative modeling approaches, including more diverse populations, and conduct cross-cultural validation to increase the generalizability of the BSFI. Longitudinal studies are also important to assess the responsiveness to changes in sexual function. These steps are essential to strengthen the evidence supporting its use in both clinical and research settings.

## Conclusion

The Brazilian version of the BSFI showed sufficient validity (content validity and hypothesis testing for construct validity) in adult men and sufficient test–retest reliability. However, its use in research and clinical practice can be limited because no model was adequate for structural validity, and the internal consistency was insufficient for at least one factor.

## Supplementary Material

BSFI_Appendix_A_qfaf029

## Appendix

### BRIEF MALE SEXUAL FUNCTION INVENTORY (BSFI) INVENTÁRIO BREVE DA FUNÇÃO SEXUAL MASCULINA

Este questionário avalia a função sexual masculina. Vamos definir o desejo sexual como um sentimento que pode incluir querer ter uma experiência sexual (masturbação ou relação sexual), pensar em fazer sexo ou sentir-se frustrado devido à falta de sexo.

1. Durante os últimos 30 dias, quantos dias você sentiu desejo sexual?
  - Nenhum dia
  - Apenas alguns dias
  - Alguns dias
  - A maioria dos dias
  - Quase todos os dias
2. Durante os últimos 30 dias, como você classificaria seu nível de desejo sexual?
  - Nenhum
  - Baixo
  - Médio
  - Alto
  - Máximo
3. Nos últimos 30 dias, a que ponto você considerou a falta de desejo sexual um problema?
  - Um problema grande
  - Um problema médio
  - Um problema pequeno
  - Um problema muito pequeno
  - Nenhum problema
4. Quanta dificuldade você teve em ter uma ereção nos últimos 30 dias?
  - Não tive nenhuma ereção
  - Muita dificuldade
  - Certa dificuldade
  - Pouca dificuldade
  - Nenhuma dificuldade
5. Nos últimos 30 dias, com que frequência você teve ereções sexuais parciais ou completas quando foi estimulado sexualmente de alguma maneira?
  - Nenhuma vez
  - Poucas vezes
  - Com certa frequência
  - Frequentemente
  - Sempre
6. Nos últimos 30 dias, quando você teve ereções, com que frequência elas foram firmes o suficiente para ter relações sexuais?
  - Nenhuma vez
  - Poucas vezes
  - Com certa frequência
  - Frequentemente
  - Sempre
7. Nos últimos 30 dias, a que ponto você considerou sua capacidade em manter uma ereção um problema?
  - Um problema grande
  - Um problema médio
  - Um problema pequeno
  - Um problema muito pequeno
  - Nenhum problema
8. Nos últimos 30 dias, quanta dificuldade você teve em ejacular quando você foi estimulado sexualmente?
  - Não tive estimulação sexual
  - Muita dificuldade
  - Certa dificuldade
  - Pouca dificuldade
  - Nenhuma dificuldade
9. Nos últimos 30 dias, a que ponto você considerou sua ejaculação um problema?
  - Um problema grande
  - Um problema médio
  - Um problema pequeno
  - Um problema muito pequeno
  - Nenhum problema
10. No geral, durante os últimos 30 dias, quão satisfeito você esteve com sua vida sexual?
  - Muito insatisfeito
  - A maioria das vezes insatisfeito
  - Neutro
  - A maioria das vezes satisfeito
  - Muito satisfeito
