# Supplementary material for: Brazilian version of the Brief Male Sexual Function Inventory (BSFI) in adult men: cultural adaptation and measurement properties
Source: Sex Med. 2025 May 12;13(2):qfaf029. doi: 10.1093/sexmed/qfaf029 (PMC12068470; doi:10.1093/sexmed/qfaf029)
Supplement: BSFI_Appendix_A_qfaf029 [file bsfi_appendix_a_qfaf029.docx]

BRIEF MALE SEXUAL FUNCTION INVENTORY (BSFI) INVENTÁRIO BREVE DA FUNÇÃO SEXUAL MASCULINA

Este questionário avalia a função sexual masculina. Vamos definir o desejo sexual como um sentimento que pode incluir querer ter uma experiência sexual (masturbação ou relação sexual), pensar em fazer sexo ou sentir-se frustrado devido à falta de sexo.

1. - Durante os últimos 30 dias, quantos dias você sentiu desejo sexual?
   - Nenhum dia
   - Apenas alguns dias
   - Alguns dias
   - A maioria dos dias
   - Quase todos os dias
2. - Durante os últimos 30 dias, como você classificaria seu nível de desejo sexual?
   - Nenhum
   - Baixo
   - Médio
   - Alto
   - Máximo
3. - Nos últimos 30 dias, a que ponto você considerou a falta de desejo sexual um problema?
   - Um problema grande
   - Um problema médio
   - Um problema pequeno
   - Um problema muito pequeno
   - Nenhum problema
4. - Quanta dificuldade você teve em ter uma ereção nos últimos 30 dias?
   - Não tive nenhuma ereção
   - Muita dificuldade
   - Certa dificuldade
   - Pouca dificuldade
   - Nenhuma dificuldade
5. - Nos últimos 30 dias, com que frequência você teve ereções sexuais parciais ou completas quando foi estimulado sexualmente de alguma maneira?
   - Nenhuma vez
   - Poucas vezes
   - Com certa frequência
   - Frequentemente
   - Sempre
6. - Nos últimos 30 dias, quando você teve ereções, com que frequência elas foram firmes o suficiente para ter relações sexuais?
   - Nenhuma vez
   - Poucas vezes
   - Com certa frequência
   - Frequentemente
   - Sempre
7. - Nos últimos 30 dias, a que ponto você considerou sua capacidade em manter uma ereção um problema?
   - Um problema grande
   - Um problema médio
   - Um problema pequeno
   - Um problema muito pequeno
   - Nenhum problema
8. - Nos últimos 30 dias, quanta dificuldade você teve em ejacular quando você foi estimulado sexualmente?
   - Não tive estimulação sexual
   - Muita dificuldade
   - Certa dificuldade
   - Pouca dificuldade
   - Nenhuma dificuldade
9. - Nos últimos 30 dias, a que ponto você considerou sua ejaculação um problema?
   - Um problema grande
   - Um problema médio
   - Um problema pequeno
   - Um problema muito pequeno
   - Nenhum problema
10. - No geral, durante os últimos 30 dias, quão satisfeito você esteve com sua vida sexual?
    - Muito insatisfeito
    - A maioria das vezes insatisfeito
    - Neutro
    - A maioria das vezes satisfeito
    - Muito satisfeito
